# A Novel Prototype African Swine Fever Virus DIVA (Differentiation Between Infected and Vaccinated Animals) Serological Assay Based on the Detection of Antibodies Against the pEP153R, eGFP, and p72 Proteins

**DOI:** 10.3390/vaccines13030211

**Published:** 2025-02-20

**Authors:** Gabriela González-García, Carmina Gallardo, Mercedes Montón, Sandra Barroso-Arévalo, Nadia Casado, José Ángel Barasona, José Manuel Sánchez-Vizcaíno, Ángel Venteo, Patricia Sastre, Paloma Rueda

**Affiliations:** 1 Gold Standard Diagnostics Madrid SA (GSD Madrid), 28037 Madrid, Spain; gabriela.gonzalezgarcia@eu.goldstandarddiagnostics.com (G.G.-G.); patricia.sastre@eu.goldstandarddiagnostics.com (P.S.); 2 European Union Reference Laboratory for African Swine Fever (EURL), Centro de Investigación en Sanidad Animal (CISA), Instituto Nacional de Investigación y Tecnología Agraria y Alimentaria (INIA), Consejo Superior de Investigaciones Científicas (CSIC), Valdeolmos, 28130 Madrid, Spain; gallardo@inia.csic.es (C.G.); nadia.casado@inia.csic.es (N.C.); 3 VISAVET Health Surveillance Center, Complutense University of Madrid, 28040 Madrid, Spain; sandrabarroso@ucm.es (S.B.-A.); jbarason@ucm.es (J.Á.B.); jmvizcaino@ucm.es (J.M.S.-V.)

**Keywords:** ASF, ASFV, DIVA, diagnosis, pEP153R, C-type lectin protein, eGFP, vaccine, ELISA

## Abstract

Background/Objectives: African Swine Fever (ASF) is one of the most significant infectious diseases affecting both domestic pig and wild boar populations, leading to substantial economic and biosanitary consequences. In Europe, disease management relies on stringent biosecurity measures and surveillance through diagnosis, highlighting the urgent need for an effective and safe vaccine for ASF control. In this context, the VACDIVA project has generated several promising vaccine candidates, including those with the EP153R gene deleted and replaced by the eGFP reporter gene. Methods: In this study, pEP153R and eGFP proteins were produced using recombinant technology and demonstrated their antigenicity and DIVA capability through indirect ELISA. Additionally, a prototype serological DIVA test was designed and developed. The assay is based on the detection of antibodies against both DIVA antigens and the well-established immunogenic p72 protein. Results: This preliminary DIVA diagnostic assay complements vaccine candidates based on a genotype II ASFV strain, featuring the deletion of the EP153R gene and/or the insertion of the eGFP reporter gene, exemplified by the Lv17/WB/Rie1-∆CD vaccine candidate. Conclusions: This approach could potentially improve surveillance during prospective vaccination campaigns.

## 1. Introduction

African Swine Fever (ASF) is an endemic disease in most countries of Sub-Saharan Africa and was successfully eradicated in Europe during the 1990s, except for the island of Sardinia, which has only recently been declared ASF-free. However, in 2007, ASF virus (ASFV) re-entered the Caucasus region of Georgia [1]. Subsequently, the virus has persisted in Eastern Europe and expanded across multiple regions on the continent, affecting both domestic pig and wild boar populations [2]. Since 2018, ASFV has made a significant incursion into new territories, with cases reported in the People’s Republic of China [3], Oceania in 2019 [4], and the Americas in 2021 [5]. In 2022, ASFV was also reported in the Italian mainland, and three new countries have notified their first occurrence of the disease: North Macedonia, Thailand, and Nepal [6]. Over the past two years, outbreaks have been reported in several new countries, including Sweden, Bosnia, Croatia, Albania, Montenegro, and Germany, reaffirming the continued spread of the virus across Europe [7].

The primary route of ASFV entry is oronasal, although alternative routes of infection have also been identified. Clinical manifestations vary depending on the virulence of the virus strain, the health status of the pig, and the animal’s age at the time of infection [8,9,10,11]. Experimental studies have shown that both the infectious dose and route of exposure play a crucial role in the onset of symptoms, which can range from high fever and lethargy to diarrhoea and haemorrhages. Four clinical forms are recognised: hyperacute, acute, subacute, and chronic. Highly virulent strains are typically associated with hyperacute or acute forms, while strains of moderate virulence may result in subacute or chronic disease. Low-virulence strains are primarily linked to chronic forms and are often associated with non-specific symptoms that can be misdiagnosed as other swine diseases. Additionally, certain animals may survive the infection and continue shedding the virus for up to 70 days post-infection, raising concerns about their potential role in ASF epidemiology [12,13].

Currently, due to the lack of a specific treatment for ASF, the approach to managing the disease is based on three key points: (1) biosecurity, (2) early identification of clinical signs, supported by laboratory diagnosis, and (3) the implementation of strict quarantine measures in the event of an outbreak [14]. These actions involve depopulation of affected farms, animal movement control and wild boar culling in the surrounding areas [15]. Consequently, the development of an effective and safe vaccine is imperative for ASF control. Thus far, the most commonly used strategies for vaccine development have involved the attenuation of wild-type ASF viruses, enhancing their safety through modification of the virus genome [16]. Several genes have been targeted so far, including those involved in immune evasion and virulence, such as CD2v/EP402R, MGF genes, UK/DP96R, or I177L [14]. Depending on the antigenicity of the proteins encoded by these modified or deleted genes, the candidate vaccine may be suitable for use in combination with a diagnostic strategy that allows for Differentiation between Infected and Vaccinated Animals (DIVA) at the serological level. In this regard, various candidate vaccines have been reported [16,17,18], and some of them, ASFV-G-∆I177L and ASFV-G-∆MGF, have even become commercially available [19,20]. However, while a few DIVA antigens have been potentially identified, companion DIVA serological tests for commercial vaccines remain elusive [21,22].

In this context, Lv17/WB/Rie1 (GenBank accession no. OR863252), a naturally attenuated non-hemadsorbing (non-HAD) strain belonging to genotype II ASFV, was isolated from a wild boar in Latvia in 2017 [23]. In vivo experiments in domestic pigs and wild boar with this isolate demonstrated potential as a vaccine [23,24]. However, since Lv17/WB/Rie1 induced subclinical signs of ASFV in experimental animals, further attenuation by gene deletion was required. These modifications could improve the vaccine safety, while maintaining potential for protection [25,26]. Some of these mutants have the C-type lectin homologue (EP153R) gene deleted and replaced by the enhanced green fluorescent protein (eGFP) reporter gene, such as Lv17/WB/Rie1-∆EP153R, Lv17/WB/Rie1-∆EP153R-∆EP402R (Lv17/WB/Rie1-∆CD) or Lv17/WB/Rie1-∆EP153R-∆EP402R-∆UK (Lv17/WB/Rie1-∆CD/UK) [27,28].

The EP153R protein (pEP153R), also known as a C-type lectin protein, is associated with the serological specificity determined by hemagglutination inhibition (HAI) [29]. Its sequence contains CD8+ T cell determinants that may potentially activate the cellular immune response against ASFV [30]. The protein is predicted to be a class II transmembrane protein with multiple glycosylation sites [31]. pEP153R has been implicated in stabilising the viral HAD phenomenon [31], although it has not been detected in viral particles [32], and its subcellular localization during infection remains undetermined. Regarding eGFP, while its antigenicity in pigs has been previously described [33,34], its role within the context of a modified ASFV remains unexplored. Despite prior assessments of the EP153R gene deletion [35,36,37] or the eGFP gene insertion [38,39] in ASF candidate vaccines, the potential DIVA properties of their gene products have not been studied before.

Hence, the objective of the present study was to produce the pEP153R and eGFP proteins in order to evaluate their antigenicity and DIVA capability in the context of an ASF infection or vaccination. Therefore, we produced a prototype DIVA serological test that detects antibodies against both target proteins and a well-established, highly immunogenic ASFV protein, such as the major capsid protein (p72) [32].

## 2. Materials and Methods

### 2.1. Bacterial Cells

One Shot™ Mach1™ T1 (Invitrogen, Waltham, MA, USA) and DH5α (Nzytech, Lisboa, Portugal) *E. coli* strains were used as host for initial cloning of target DNA and for maintaining plasmids. One Shot™ BL21 *E. coli* (DE3) strain (Invitrogen, Waltham, MA, USA) was used as the bacterial host for protein expression. The bacterial strains were grown at 37 °C and 225 rpm in Luria-Bertani (LB) medium with the corresponding antibiotic for each plasmid.

### 2.2. Mammalian Cells

HEK-293-F cells (Invitrogen, Waltham, MA, USA) were grown in Freestyle™ 293 medium (Thermo Fisher Scientific, Waltham, MA, USA) and maintained at 37 °C, 135 rpm and 8% CO_2_ atmosphere.

### 2.3. Sera

Five panels of experimental sera were used in this study (Table 1). Sera from domestic pigs were from experiments conducted at Biosafety Level 3 (BSL-3) facilities at CISA-INIA/CSIC (Valdeolmos, Madrid, Spain), and sera from wild boar were collected at BSL-3 facilities at VISAVET, UCM (Madrid, Spain). All the in vivo experiments were conducted in accordance with the EC Directive 86/609/EEC, Spanish Ethical and Animal Welfare Committee (PROEX 125/16, 159/19, 113.3/21, 090.4/21 and 101.8/21). The domestic pigs originated from the same farm and were confirmed to be ASFV-free and free of ASFV antibodies. These were 12-week-old European hybrid pigs weighing between 20 and 25 kg. The wild boar consisted of 3- to 4-month-old female piglets weighing 10 to 15 kg, obtained from a commercial wild boar farm in Extremadura, Spain.

All ASFV isolates and vaccine strains used in the in vivo experiments were propagated in primary cultures of porcine alveolar macrophages (PAMs) or peripheral blood monocytes (PBMs). The titration of non-HAD ASFV strains and modified vaccine candidates was performed on Cos7 (ATCC CRL-1651) or PAMs cells, respectively, with viral titres expressed as the quantity of virus inducing a cytopathic effect in 50% of infected cultures (TCID_50_), as assessed by immunoperoxidase staining [40].

The seroconversion of all the animals included in this study against ASFV was confirmed by a commercial ELISA based on p72 (INgezim^®^ PPA Compac, GSD Madrid SA, Madrid, Spain).

- *Group A1: Domestic pigs infected with the parental virus Lv17/WB/Rie1.* Forty-three sera were derived from 5 domestic pigs experimentally inoculated by intramuscular (IM) route, with 10 or 10^2^ TCID_50_ of Lv17/WB/Rie1. The five animals were challenged at 30 days post-infection (dpi) [27,28]. Additionally, we included 72 samples from two other pigs, one inoculated with 10 TCID_50_ of Lv17/WB/Rie1 (PW13) and the other put in contact (PW14) with animal PW13. Then, these last two animals were put in contact with the virulent strain Lv17/WB/Zieme3 at 58 dpi. The sera analysed were collected between 0 and 126 dpi [23].
- *Group A2: Domestic pigs vaccinated with three vaccine candidates.* One hundred and ninety-seven serum samples were derived from 16 domestic pigs experimentally vaccinated by the IM route, with 10^2^ TCID_50_ of different prototype marker vaccines (Lv17/WB/Rie1-∆EP153R, Lv17/WB/Rie1-∆CD, Lv17/WB/Rie1-∆CD/UK). All these animals were challenged at 30 or 35 dpi. The sera analysed were collected between 0 and 54 days post-vaccination (dpv) [27,28].
- *Group B1: Wild boar immunised with the parental virus Lv17/WB/Rie1.* One hundred and seven sera were derived from 8 wild boar experimentally immunised by the oral route with 10^3^ or 10^4^ TCID_50_ of Lv17/WB/Rie1 and revaccinated with the same dose at 18 days post-prime vaccination. The samples were collected between 0 and 89 dpi. All the animals were challenged at 42 dpi [24].
- *Group B2: Wild boar vaccinated with the vaccine candidate Lv17/WB/Rie1-ΔCD.* Ninety-six sera were derived from 8 wild boar experimentally vaccinated by the oral route. Four out of these eight animals were vaccinated with 10^2^ TCID_50_ of Lv17/WB/Rie1-ΔCD, boosted 30 days later with 10^4^ TCID_50_ of the same vaccine candidate, and challenged 14 days after the booster. On the other hand, the other 4 animals were vaccinated with 10^4^ TCID_50_ of Lv17/WB/Rie1-ΔCD and challenged at 30 dpv. Sera were collected between 0 and 62 dpv [27].
- *Group C: Domestic pigs infected with other ASFV isolates, belonging to genotype II and different from the parental virus.* One hundred and two sera were derived from 4 domestic pigs experimentally inoculated by the IM route. Two out of the four were infected with 10 TCID_50_/mL Pol18/WB/Case1794, and the other two with the same dose of the Lv17/WB/Rie14/Tukuma5. Sera were collected between 0 and 121 dpi [unpublished EURL data].

The challenges were performed via the IM route, with a dose of 10 50% hemadsorbing units (HAU_50_) of Arm07, a genotype II highly virulent ASFV strain, whose reliability and effectiveness have been previously reported [41]. This challenge was applied to all animals, except for 2 out of the 7 animals in Group A1, which were challenged as previously described.

Furthermore, 384 serum samples collected from animals in ASF-free Spanish areas were also included in the analysis: 150 were from pigs and 234 from wild boar.

### 2.4. Production and Characterisation of the Recombinant Proteins

The nucleotide sequence coding for the completed extracellular domain of the pEP153R of Lv17/WB/Rie1 strain was amplified from a synthetic gene (Eurofins Genomics, Ebersberg, Germany) optimised for *Homo sapiens* using specific forward and reverse primers (Table 2). This DNA fragment was cloned into the vector pCMV6-AC-FC-S (Origene, Rockville, MA, USA) by double digestion with AscI and XhoI enzymes (Thermo Fisher Scientific, Waltham, MA, USA) according to the manufacturer’s instructions. This vector adds an interleukin-2 secretion signal to the 5′-end and a mouse Fc tag to the 3′-end of the gene. The sequence of the recombinant plasmid was confirmed by Sanger sequencing. The plasmid was used to transfect HEK-293-F cells, following the FectoPRO^®^ reagent protocol (Polyplus, Illkirch-Graffenstaden, France). At 6 days post-transfection, the culture was harvested and pEP153R was recovered from the culture medium and purified by protein G affinity chromatography as described previously [42].

The protein was analysed by SDS-PAGE with Coomassie staining, using the Precision Plus Protein Dual Color Standards (BioRad, Alcobendas, Spain) as the molecular weight ladder. Once the gel was stained, protein quantification was carried out by densitometry, using a standard curve with known amounts of BSA (0.5, 1.0, and 1.5 µg) and the image analysis software TotalLab 1D, version 14.0 (TotalLab, Newcastle upon Tyne, England). The purity level was also determined using this programme. On the other hand, the protein identity was confirmed by immunoblotting. In this technique, the protein was transferred to PVDF membranes, followed by blocking with 5% milk in PBST for 1 h at RT. The membranes were probe with 1:5000 dilution of goat anti-mouse IgG (H + L) monoclonal antibody (mAb), HRP (Invitrogen, Waltham, MA, USA). The membrane was washed 3 times with PBST and detected with ep (HS)TMB-mA (SDT, Baesweiler, Germany).

The full-length sequence of the eGFP was expressed in a bacterial expression system. The nucleotide sequence was amplified from a synthetic gene optimised for expression in *E. coli* (Eurofins Genomics, Ebersberg, Germany) using specific forward and reverse primers (Table 2). The gene was cloned into the pDEST™15 vector (Thermo Fisher Scientific, Waltham, MA, USA), which adds a GST tag to the 5′-end of the gene, using the reagents and following the protocol of the Gateway™ cloning system (Thermo Fisher Scientific, Waltham, MA, USA). The sequence of the recombinant plasmid was confirmed by Sanger sequencing. The plasmid was used to transform *E. coli* cells by heat shock. Bacteria were grown and the protein expression was induced as described previously [43]. The culture was maintained for 18 h at 18 °C, 225 rpm. eGFP was obtained from the soluble fraction of the cellular extract and purified by affinity chromatography, using a GST GraviTrap column (GE Healthcare, Madrid, Spain). The protein was evaluated by SDS-PAGE with Coomassie staining, following the same protocol described above for pEP153R analysis. The protein identity was confirmed by immunoblotting, using an HRP-conjugated mouse mAb against the GST tag (3H11, GSD Madrid SA, Madrid, Spain).

### 2.5. ELISA

Microtitre plates (Corning, Somerville, MA, USA) were coated with 15 ng/well of pEP153R or 5 ng/well of eGFP, in 0.05 M sodium carbonate buffer, pH 9.6, and incubated overnight at 4 °C. After the plates were blocked with StabilZyme SELECT^®^ Stabilizer (SS, Surmodics, Eden Prairie, MI, USA) for 1 h at RT, the wells were incubated with the swine serum diluted 1:100 in serum dilution buffer (DE34, GSD Madrid SA, Madrid, Spain) for 1 h at RT. An HRP-conjugated anti-swine mouse IgG mAb (1BH7, GSD Madrid SA, Madrid, Spain) was used subsequently as secondary antibody for 1 h at RT. TMB (Enhanced K-blue TMB, Neogen, Scotland, UK) was added, and the reaction was stopped 20 min later by adding 0.5 M sulfuric acid. Enzymatic activity was measured by determination of the optical density (OD) at 450 nm in an ELISA plate reader. Washes between consecutive steps were performed with 0.05% Tween 20 in phosphate-buffered saline.

Additionally, non-related antigens, referring to those not derived from viruses that affect swine, were expressed in the same system and under the same conditions as each target protein (negative antigens). These proteins were also coated on the plates, using the same protocol previously described for pEP153R and eGFP.

The results from the ELISAs were expressed as the OD value for each sample (S) minus the mean of a negative control (NC) run in duplicate (S-NC).

### 2.6. Statistical Analysis

Data were statistically analysed by a ROC analysis (95% confidence interval, CI) using MedCalc^®^ software (version 10.1.7.0) to establish the optimal cut-off value as well as the sensitivity and specificity parameters for each newly developed assay. For the statistical evaluation, samples were classified as positive or negative according to INgezim^®^ PPA Compac (GSD Madrid SA, Madrid, Spain). In this context, all vaccinated animals were considered negative by pEP153R ELISA, and infected animals were considered negative by eGFP ELISA. Furthermore, to provide a visual representation of the distribution of the data, box-and-whisker plots were performed.

## 3. Results

### 3.1. Production and Characterisation of the Recombinant Proteins

**Figure 1:**
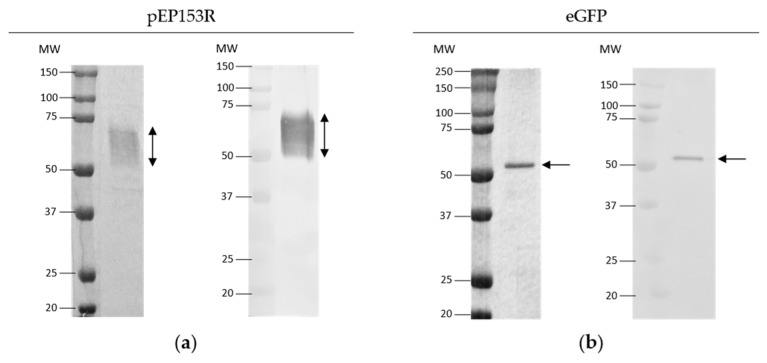
Analysis of the purified recombinant proteins. (**a**) pEP153R; (**b**) eGFP. In each case, the left panel shows the analysis by SDS-PAGE with Coomassie staining, and the right panel shows the analysis by immunoblotting using a mouse monoclonal antibody against the tag of each protein. Arrows indicate the band corresponding to the protein of interest. The numbers on the left side of the gels indicate the molecular weight (MW) of each band in the protein ladder (kDa).

The purity and molecular weight of the recombinant purified pEP153R and eGFP proteins were analysed by SDS-PAGE with Coomassie staining and their identity by immunoblotting, using a mAb against their corresponding tag (Figure 1). A band with the expected molecular weight was detected for each protein in both analyses: approximately 70 kDa for pEP153R-Fc, considering its predicted N-glycosylation sites [31], and 55 kDa for GST-eGFP. The purity of the proteins produced was approximately 94% for pEP153R and 95% for eGFP.

**Figure 2:**
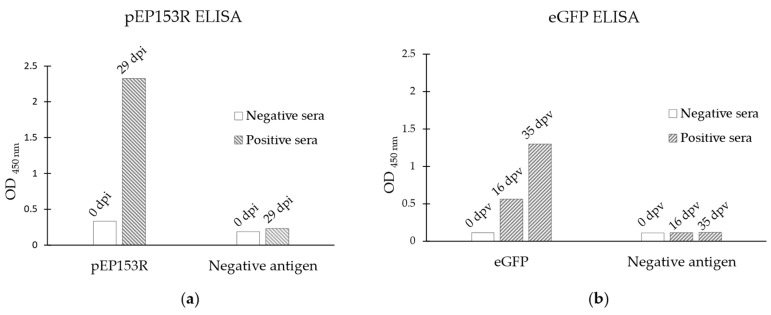
Analysis of the antigenicity of the target proteins by indirect ELISA: (**a**) pEP153R, using negative and positive serum samples from a domestic pig infected with Lv17/WB/Rie1; (**b**) eGFP, analysing negative and positive serum samples from a domestic pig vaccinated with Lv17/WB/Rie1-ΔEP153R. In each graph, the y-axis shows the optical density (OD) obtained in the assay, and the x-axis indicates the antigen coated on the plate. dpi/dpv: days post-infection/vaccination.

The antigenicity of the proteins was analysed by indirect ELISA using sera from Groups A1 or A2, collected at different dpi or dpv, respectively. A differential signal between negative (0 dpi/dpv) and positive (16, 29, and 35 dpi/dpv) sera was observed for both assays. Furthermore, when the same sera were analysed against negative antigens, no signal was detected in any of the assays (Figure 2).

### 3.2. Prototype Indirect ELISAs

In order to establish the best conditions for the pEP153R and eGFP ELISAs, optimisation experiments were performed on antigen-coated concentrations, serum dilutions, and HRP-conjugated mouse anti-swine IgG mAb dilutions. Additionally, the antibody response against p72 (INgezim^®^ PPA Compac, GSD Madrid SA, Madrid, Spain) was used in this study as a control of the infection and for monitoring immunity in vaccinated animals.

**Figure 3:**
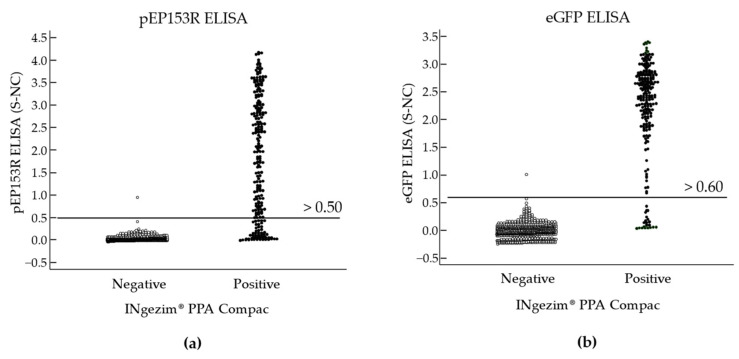
Interactive dot diagram (ROC analysis) for the indirect ELISAs: (**a**) ELISA pEP153R; (**b**) ELISA eGFP. The horizontal line indicates the cut-off value for each assay (S-NC > 0.50 and S-NC > 0.60, respectively). S-NC: sample OD minus the OD of the negative control of the assay.

Once the eGFP and pEP153R ELISA conditions mentioned above were optimised, several serum samples from domestic pigs and wild boar were analysed to evaluate the DIVA characteristics of both antigens. As shown in Figure 3, the cut-off value of the assays was defined as 0.5 for pEP153R ELISA and 0.6 for eGFP ELISA, considering all the samples analysed.

#### 3.2.1. Analysis of Sera from Domestic Pigs Experimentally Immunized with Lv17/WB/Rie1 or the Candidate Vaccines Lv17/WB/Rie1-∆EP153R, Lv17/WB/Rie1-∆CD, Lv17/WB/Rie1-∆CD/UK

**Figure 4:**
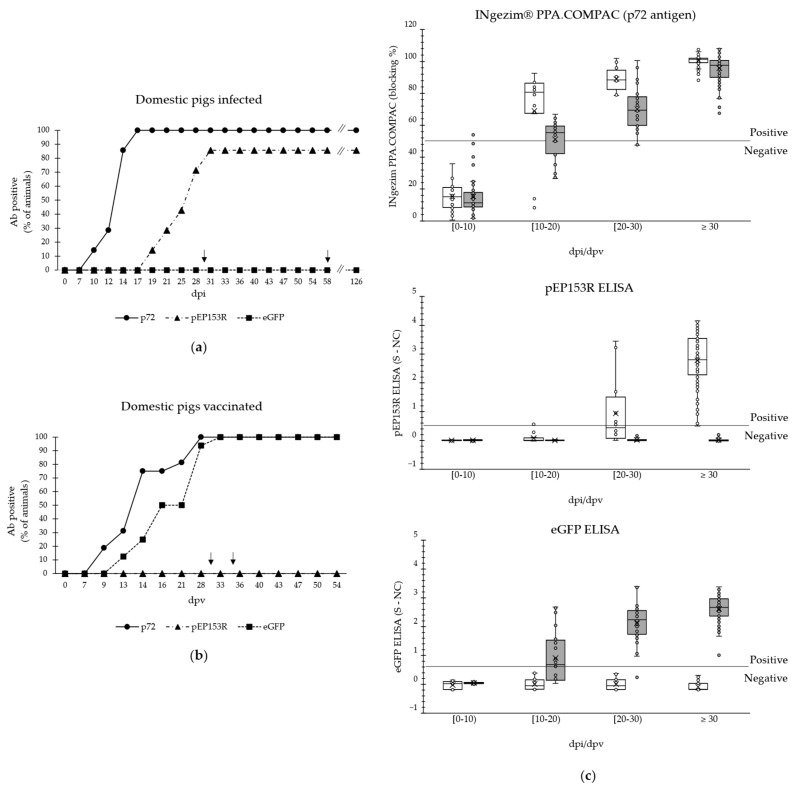
Analysis of the antibody (Ab) response in domestic pigs against the antigens p72 (INgezim^®^ PPA Compac), pEP153R (pEP153R ELISA), and eGFP (eGFP ELISA). (**a**) Animals infected with Lv17/WB/Rie1; (**b**) animals vaccinated with different prototype vaccines; (**c**) data classified by ELISA, from infected (white boxes) or vaccinated (grey boxes) animals. The cut-off of each assay is indicated by a horizontal line. In (**a**,**b**), the y-axis shows the percentage of Ab-positive animals in each assay; and in (**c**), the ELISA blocking percentage or S-NC parameter. In all cases, the x-axis indicates the days post-infection/vaccination (dpi/dpv). The arrows indicate the day on which the animals were challenged (at 30, 35, or 58 dpi/dpv).

A panel of 115 serum samples from 7 animals of Group A1 and 207 samples from 16 animals of Group A2 was analysed (Table 3). As Figure 4a shows, 100% of the animals infected with the parental virus developed an antibody response against p72 at 14 ± 2 dpi and 86% developed one against pEP153R at 25 ± 5 dpi. The animal that did not seroconvert against pEP153R died at 12 dpi. Samples from animals PW13 and PW14 remained positive against pEP153R until 58 dpi (before exposure to the genotype II virulent ASFV strain), and the level of antibodies was maintained until the last sample collected at 68 days post-challenge. On the other hand, all the animals remained negative against eGFP.

Regarding vaccinated pigs, 100% seroconverted against p72 at 15 ± 4 dpv and against eGFP at 18 ± 4 dpv while remaining negative against pEP153R until the last sample analysed, collected at 54 dpv (Figure 4b).

The quantitative data, clustered by dpi, obtained from the three ELISAs performed with samples from infected and vaccinated animals are represented in Figure 4c. Regarding the pPE153R ELISA, a sensitivity of 10.0% [95% CI: 1.7% to 44.5%] was detected under 20 dpi, increasing to 50.0% [95% CI: 21.2% to 78.8%] for the subsequent 10 days and finally reaching 100.0% [95% CI: 95.2% to 100.0%] for samples collected at 30 dpi or later. In respect to the eGFP assay, for the same time intervals, the sensitivity was of 58.3% [95% CI: 36.7% to 77.9%], 96.9% [95% CI: 83.7% to 99.5%], and 100.0% [95% CI: 96.1% to 100.0%], respectively. The specificity of the newly developed ELISAs was of 100.0% for both assays [95% CI: 98.4% to 100.0% for pEP153R assay and 97.7% to 100.0% for eGFP], considering all the samples analysed.

#### 3.2.2. Analysis of Sera from Wild Boar Experimentally Immunised with Lv17/WB/Rie1 or the Candidate Vaccine Lv17/WB/Rie1-∆CD

**Figure 5:**
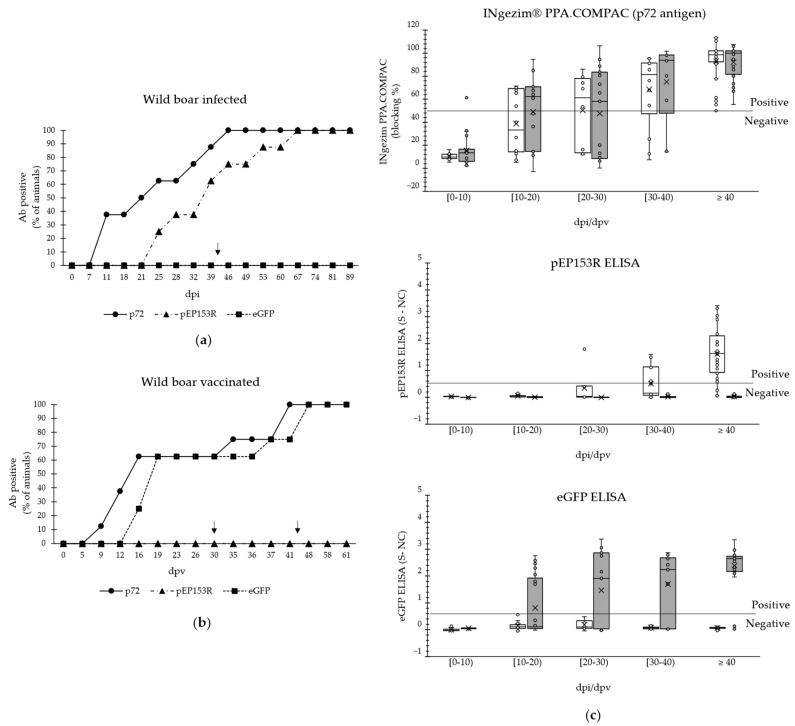
Analysis of the antibody (Ab) response in wild boar against the antigens p72 (INgezim^®^ PPA Compac), pEP153R (pEP153R ELISA), and eGFP (eGFP ELISA). (**a**) Animals infected with Lv17/WB/Rie1; (**b**) animals vaccinated with Lv17/WB/Rie1-∆CD; (**c**) data classified by ELISA, from infected (white boxes) or vaccinated (grey boxes) animals. The cut-off of each assay is indicated by a horizontal line. In (**a**,**b**), the y-axis shows the percentage of Ab-positive animals in each assay; and in (**c**), the ELISA blocking percentage or S-NC parameter. In all cases, the x-axis indicates the days post-infection/vaccination (dpi/dpv). The arrows indicate the day on which the animals were challenged (30, 42, or 44 dpi/dpv).

The samples from Group B1 and B2 were analysed (Table 4). The seroconversion of all the experimentally immunised or vaccinated wild boar was confirmed using INgezim^®^ PPA Compac. As Figure 5 shows, the serum samples were re-analysed with the same commercial assay for the purpose of this study. Regarding animals immunised with the parental virus Lv17/WB/Rie1, 100% of them developed an antibody response against pEP153R at different times after 25 dpi while remaining negative against eGFP (Figure 5a). Regarding wild boar vaccinated with the mutant strain, all the animals seroconverted against eGFP at different times after 16 dpv and remained negative against pEP153R, even after the challenge with the Arm07 strain (Figure 5b).

The quantitative data from the three ELISAs, organised by dpi, are presented in Figure 5c and were obtained using samples from infected and vaccinated wild boar. In the pPE153R ELISA, the sensitivity was initially 0.0% [95% CI: 0.0% to 41.1%] for samples collected under 20 dpi and increased to 20.0% [95% CI: 3.3% to 71.2%] for the following 10 days and to 54.6% [95% CI: 23.5 to 83.1] for the [30–40) dpi time interval. The sensitivity reached 87.9% [95% CI: 71,8% to 96.5%] for samples collected at 40 dpi or later. Similarly, the eGFP assay showed a sensitivity of 50.0% [95% CI: 21.2% to 78.8%], 100% [95% CI: 69.0% to 100.0%], 87.5% [95% CI: 47.4% to 97.9%], and 92.9% [95% CI: 76.5% to 98.9%] for the same time intervals. Based on all analysed samples, the specificity of both newly developed ELISAs was of 100.0%, with a 95% CI of 97.0% to 100.0% for the pPE153R assay and 96.9% to 100.0% for the eGFP ELISA.

#### 3.2.3. Sera from Field Animals

In order to study the specificity of the prototype DIVA indirect ELISAs, a panel of 384 field samples from ASF-free areas were assessed against pEP153R and eGFP: 150 sera were from pigs and 234 sera were from wild boar. In both ELISAs, diagnostic specificity was 100.0% [95% CI: 97.6% to 100.0%] for pigs and 99.6% [95% CI: 97.6% to 99.9%] in the case of wild boar.

#### 3.2.4. Sera from Domestic Pigs Experimentally Infected with Other Genotype II ASFV Isolates

**Figure 6:**
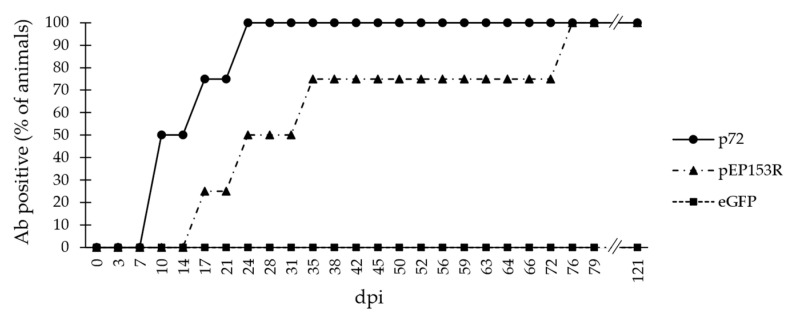
Antibody (Ab) response of domestic pigs experimentally infected with other attenuated genotype II ASFV isolates, different from the parental virus Lv17/WB/Rie1. The y-axis shows the percentage of Ab-positive animals against the proteins p72 (INgezim^®^ PPA Compac), pEP153R (pEP153R ELISA), or eGFP (eGFP ELISA), and the x-axis shows the days post-infection (dpi).

To evaluate the immunogenicity of the pEP153R antigen in infections caused by other attenuated genotype II ASFV isolates, 102 serum samples from two domestic pigs experimentally infected with Pol18/WB/Case1794 and two animals inoculated with Lv17/WB/Rie14/Tukuma5 (Group C) were evaluated. All the animals analysed developed an antibody response against p72 at different times after 15 ± 7 dpi, as well as to pEP153R, with a delay which varies depending on the animal. The signal against both antigens remained positive until the last sample collected at 121 dpi. The pPE153R ELISA showed a sensitivity of 81.8% [95% CI: 70.4% to 90.2%] for samples collected at 30 dpi or later and a specificity of 100.0% [95% CI: 81.3% to 100.0%] considering all the samples. On the other hand, all the animals remained negative against eGFP (specificity of eGFP ELISA: 100.0% [95% CI: 96.4% to 100.0%]) (Figure 6).

## 4. Discussion

The urgency to contain ASF disease emphasises the pivotal role of developing an effective and safe vaccine for disease control [6,7]. Molecular and serological DIVA characteristics are integral for an ASF vaccine to enable effective surveillance in regions where vaccination campaigns will be carried out [44]. However, until now, the ASF DIVA diagnosis approach is usually limited to time intervals when viremia is detected [45,46], although there are a few studies evaluating serological DIVA performance, including a reduced number of samples. These emerging studies are limited by small sample sizes and the use of pooled samples without analysing the kinetics of seroconversion against the antigens [14,21,22,47,48,49].

In the context of achieving an ASF DIVA vaccine, several mutants have been generated under VACDIVA project. Some of these candidates, based on Lv17/WB/Rie1 parental virus, have the EP153R gene deleted and replaced by the eGFP reporter gene [26,27]. In the present study, the proteins encoded by these two target genes were produced. Subsequently, their potential as DIVA antigens was assessed using a newly developed serological assay based on the detection of antibodies against both target antigens in pigs and wild boar. Furthermore, a highly immunogenic viral antigen was included in the DIVA test to control the infection and monitoring vaccination. As described previously, p72 was selected in this study since it is the major capsid protein of ASFV, is highly conserved among isolates, and is one of the most immunogenic proteins of the virus [50]. However, other well-characterised immunogenic ASFV proteins, such as p30 [51], could also be considered for the assay.

The results obtained in the present study showed that all domestic pigs inoculated experimentally with the parental virus Lv17/WB/Rie1 or other wild-type genotype II ASF viruses (Pol18/WB/Case1794 and Lv17/WB/Rie14/Tukuma5) seroconverted against p72. If the pig that died at 12 dpi is not considered, all the animals subsequently developed an antibody response against pEP153R (Figure 4a), with an onset delay that varies depending on the animal, with an average of 2 weeks. The assay showed a sensitivity of over 90% for samples collected at 30 dpi or later. This result highlights the need for continuous monitoring of p72 seroconversion as a control. This limitation was expected, as pEP153R is a non-essential antigen for virus replication and has not been previously characterised as one of the primary immunogenic proteins of the virus. However, in all cases, the signal against pEP153R remained positive until the last sample collected (at 126 dpi for 2 animals). The antibody response detected against pEP153R seems to be more heterogeneous compared to the seroconversion against p72 (Figure 4c). This phenomenon may be an implicit consequence in the onset of the two antibody responses. Furthermore, as expected, all the samples from these infected animals were negative to eGFP (Figure 4a), resulting in a specificity of 100%.

On the other hand, even after the challenge with a virulent genotype II ASFV strain, all samples from vaccinated pigs were negative against pEP153R until the last day analysed (54 dpv). However, further analysis of additional samples collected over an extended period is required to determine whether the antibody titre increases beyond this time point. This investigation would be particularly relevant in animals where viremia was detected following the challenge, as described by Carmina et al., 2024 [28]. Furthermore, all vaccinated pigs produced a high level of antibodies against eGFP, which is maintained until the last sample analysed at 54 dpv. The antibody profile for eGFP was very similar to the response against p72 control protein, within a time frame of 4 ± 4 days between both (Figure 4b), which is reflected in sensitivity values of up to 100% for samples collected at 30 dpi or later. These results support the potential application of eGFP as DIVA antigen.

Regarding wild boar, the results observed were very similar to the ones obtained for domestic pigs: all the animals immunised with Lv17/WB/Rie1 virus seroconverted against pEP153R at different times post-infection, maintaining the level of antibodies until the last sample analysed (89 dpi). As with domestic pigs, this response presented a delayed start of approximately 2 weeks compared to the appearance of antibodies against p72, which is reflected in the sensitivity values. Regarding eGFP antibodies, all the infected wild boar remained negative (Figure 5a). It is important to note that the humoral response against the three targets of the DIVA assay was more homogeneous in domestic pigs (Figure 4c) than in wild boar (Figure 5c). The differences between the two animals could be due to the fact that, in the case of wild boar, two different doses of immunisation were tested together, which could have increased the variability in the responses. This is consistent with previous observations indicating that the appearance of antibodies is related to the dose used [8,9,10,11]. On the other hand, all samples from vaccinated wild boar were negative against pEP153R even after the challenge with Arm07. These vaccinated animals also developed a similar antibody profile against p72 and eGFP, within a time window of 5 ± 3 days between both (Figure 5b), showing a sensitivity of 93% for samples collected at 40 dpi or later.

In this context, previous evidence suggested that pEP153R may play a role in HAI serological specificity [29] and in the cellular immune response against ASFV [30]. Although its immunogenicity has not been definitively established [52], recent studies have indicated its capability to trigger specific antibodies in the context of an ASFV infection [53]. However, to our knowledge, the present study represents the first experimental report about the kinetics of a specific humoral immune response against pEP153R. Regarding eGFP, although its immunogenicity has been previously described in pigs [33,34], it has not been evaluated before within the context of a modified ASFV. Additionally, the results presented here indicate, for the first time, that pEP153R and eGFP could be used as antigens for the DIVA diagnosis of ASF in domestic pigs and wild boar.

This newly developed prototype DIVA serological assay could accompany an ASF vaccine that incorporates the deletion of the EP153R gene and the insertion of the eGFP reporter gene, such as Lv17/WB/Rie1-∆CD vaccine candidate [27]. Since pEP153R is genotype-specific, the use of this novel serological DIVA assay could be included in conventional ASF surveillance in potentially vaccinated areas in Europe, America, and Asia, since the majority of ASFV isolates circulating in those regions corresponds to genotype II strains [5,54,55]. Thus, the assay could be used for monitoring the immunisation level of vaccinated animals, in farms or wildlife populations. These vaccinated animals will be positive by eGFP ELISA and INgezim^®^ PPA Compac (or another conventional diagnostic assay [50]) and will be negative by pEP153R ELISA. The similar sensitivity of the eGFP ELISA and the INgezim^®^ PPA Compac assay, as well as the good specificity of both novel indirect ELISAs in experimental animals, will allow for a reliable DIVA diagnosis. Nevertheless, future studies should include the evaluation of field samples from animals with natural infections to assess the performance of the assays under real-world conditions.

However, if signs associated with ASF appear on a farm or in the field, the DIVA diagnosis could differentiate between the vaccine and wild-type ASFV strains. In cases of peracute or acute forms of the disease, the affected animals typically do not produce antibodies against ASFV [56]. Thus, the use of the DIVA PCR accompanying the vaccine becomes imperative [57]. In contrast, some animals may develop a subacute or chronic ASF, or even remain subclinically infected, particularly when infected by low virulent strains [58,59]. In these last cases, analysing the antibody response against ASF is crucial for surveillance, and consequently, a serological DIVA performance of the vaccine will be required. Nevertheless, as described in the present study, the antibody kinetics against pEP153R appears later than the response against p72 control protein, exhibiting an approximate delay of 2 weeks. Therefore, earlier antibody detection, such as the one against p72, becomes essential in DIVA serological diagnosis to control ASFV infections. However, if the infected animal survives this time window, the results presented here suggest the subsequent development of an antibody response against pEP153R. Thus, sera from these animals will be positive by INgezim^®^ PPA Compac and pEP153R ELISA and negative by eGFP ELISA, which indicates that the animal was infected by a genotype II ASFV strain. The interpretation of the DIVA serological assay for a given sample, based on the results obtained from the three independent ELISAs, is summarised in Table 5, considering the significant variability of pEP153R among genotypes [29].

Additionally, between both antibody onsets against p72 and pEP153R (approximately 2 weeks), the DIVA PCR [57] will play an essential role in DIVA surveillance. For these reasons, the combination of both serological and molecular DIVA diagnostic tests becomes crucial for the design of future vaccination protocols, especially to detect ASFV infections in early stages.

Finally, although this work represents the first report with an extensive dataset and a detailed evaluation of seroconversion kinetics in individual animals, analysing a larger number of animals in field trials is necessary to further validate the findings and enhance the robustness of the conclusions.

## 5. Conclusions

The data presented in this study describe a serological DIVA test based on the ELISA platform. For all genotype II ASFV-infected animals analysed, a seroconversion against pEP153R was observed, although with a delayed onset compared to p72 control protein, while remaining negative against eGFP. On the other hand, 100% of vaccinated animals included in this study exhibited the opposite antibody pattern, since all the animals seroconverted against the reporter protein and remained negative against pEP153R. This performance makes this assay a potential tool for surveillance, particularly in regions where a vaccine incorporating the deletion of the EP153R gene and/or the insertion of the eGFP reporter gene could be applied.

## 6. Patents

The results of this research are protected by the patent “Method for Differentiating ASFV Infected from ASFV Vaccinated Animals” (not yet published; application number: PCT/EP2023/082521).

## Figures and Tables

**Table 1:** Experimental sera used in this study.

Sample Group	ASFV Strain or Vaccine	Type of Animal	Route	Dose (TCID_50_)	Nº of Samples	Nº of Animals	dpi/ dpv	Sampling Interval *
A1	Lv17/WB/Rie1	DP	IM	10^2^	43	5	0–54	7 days
			IM	10	36	1	0–126	3–4 days
			ICP	-	36	1	0–126	3–4 days
A2	Lv17/WB/Rie1-ΔEP153R	DP	IM	10^2^	77	6	0–54	7 days
	Lv17/WB/Rie1-ΔCD			10^2^	60	5	0–54	7 days
	Lv17/WB/Rie1-ΔCD/UK			10^2^	60	5	0–54	7 days
B1	Lv17/WB/Rie1	WB	Oral	10^3^ + 10^3^	62	5	0–89	7 days
				10^4^ + 10^4^	45	3	0–89	7 days
B2	Lv17/WB/Rie1-ΔCD	WB	Oral	10^2^ + 10^4^	52	4	0–62	7 days
				10^4^	44	4	0–61	7 days
C	Pol18/WB/Case1794	DP	IM	10	40	2	0–66	3–4 days
	Lv17/WB/Rie14/Tukuma5			10	62	2	0–121	3–4 days

* Time between samples analysed. Lv17/WB/Rie1-ΔCD: Lv17/WB/Rie1/WB/Rie1-ΔEP153R-ΔEP402R; Lv17/WB/Rie1-ΔCD/UK: Lv17/WB/Rie1/WB/Rie1-ΔEP153R-ΔEP402R-ΔUK. DP: domestic pig; WB: wild boar; IM: intramuscular; ICP: in contact pig; dpi/dpv: days post-infection/vaccination.

**Table 2:** Specific nucleotide primers used in EP153R and eGFP cloning.

	Forward ^1^	Reverse ^1^
pEP153R	5’ TTGGCGCGCCaataaataaacccatatgt	5’CCGCTCGAG*ACCTTGGAAATAGAGATTTTC*cttgctgcatatgtagagcaa
eGFP	5′atggtgagcaaaggtgaagaactg	5′ttatttgtacagttcatccatacctaag

^1^ Complementary sequences of the genes are indicated in lowercase; the restriction sites are shown underlined; TEV protease recognition sequence is indicated in italic font (this sequence was included to remove the Fc tag if it interfered with the immunoassays).

**Table 3:** Serum samples from domestic pigs analysed by both prototype indirect ELISAs and INgezim^®^ PPA Compac.

Domestic Pigs	Group A1				Group A2			
**dpi/dpv**	[0–10)	[10–20)	[20–30)	≥30	[0–10)	[10–20)	[20–30)	≥30
**Animal No.**	7	7	5	5	16	16	14	14
**Sample No.**	16	12	12	75	44	28	32	93

**Table 4:** Serum samples from wild boar analysed by the prototype indirect ELISAs and INgezim^®^ PPA Compac.

Wild Boar	Group B1					Group B2				
**dpi/dpv**	[0–10)	[10–20)	[20–30)	[30–40)	≥40	[0–10)	[10–20)	[20–30)	[30–40)	≥40
**Animal No.**	7	8	8	7	8	8	8	7	7	8
**Sample No.**	13	15	9	14	56	20	19	19	10	28

**Table 5:** Interpretation of the serological DIVA assay.

**Interpretation**	**ELISA Results Against Each Target Protein**		
	**p72**	**pEP153R**	**eGFP**
Vaccinated *	P	N	P
Infected with a genotype II ASFV strain	P	P	N
Infected with a ASFV strain belonging to other genotype different from II or the time frame between p72 and pEP153R/eGFP antibody responses	P	N	N
ASFV non-infected or an early stage of the infection	N	N	N

P: positive; N: negative. * The results would be the same for animals infected after receiving the vaccine.

## Data Availability

The original contributions presented in this study are included in the article; further inquiries can be directed to the corresponding author.

## References

[1] Rowlands R.J., Michaud V., Heath L., Hutchings G., Oura C., Vosloo W., Dwarka R., Onashvili T., Albina E., Dixon L.K. (2008). African Swine Fever Virus Isolate, Georgia, 2007. Emerg. Infect. Dis..

[2] Sánchez-Vizcaíno J.M., Mur L., Martínez-López B. (2013). African Swine Fever (ASF): Five Years around Europe. Vet. Microbiol..

[3] Zhou X., Li N., Luo Y., Liu Y., Miao F., Chen T., Zhang S., Cao P., Li X., Tian K. (2018). Emergence of African Swine Fever in China, 2018. Transbound. Emerg. Dis..

[4] Barnes T.S., Morais O., Cargill C., Parke C.R., Urlings A. (2020). First Steps in Managing the Challenge of African Swine Fever in Timor-Leste. One Health.

[5] Gonzales W., Moreno C., Duran U., Henao N., Bencosme M., Lora P., Reyes R., Núñez R., De Gracia A., Perez A.M. (2021). African Swine Fever in the Dominican Republic. Transbound. Emerg. Dis..

[6] WOAH (2022). African Swine Fever (ASF)—Situation Report 11. https://www.woah.org/app/uploads/2022/06/asf-report11.pdf.

[7] WOAH World Animal Health Information System (WAHIS). https://wahis.woah.org/#/home.

[8] Sánchez-Cordón P.J., Vidaña B., Neimanis A., Núñez A., Wikstrom E., Gavier-Widén D. (2021). Pathology of African Swine Fever. Understanding and Combatting African Swine Fever: A European Perspective.

[9] Salguero F.J. (2020). Comparative Pathology and Pathogenesis of African Swine Fever Infection in Swine. Front. Vet. Sci..

[10] Sánchez-Cordón P.J., Chapman D., Jabbar T., Reis A.L., Goatley L., Netherton C.L., Taylor G., Montoya M., Dixon L. (2017). Different Routes and Doses Influence Protection in Pigs Immunised with the Naturally Attenuated African Swine Fever Virus Isolate OURT88/3. Antivir. Res..

[11] Vlasov M., Sindryakova I., Kudryashov D., Morgunov S., Kolbasova O., Lyska V., Zhivoderov S., Pivova E., Balyshev V., Namsrayn S. (2024). Administration Routes and Doses of the Attenuated African Swine Fever Virus Strain PSA-1NH Influence Cross-Protection of Pigs against Heterologous Challenge. Animals.

[12] Ståhl K., Sternberg-Lewerin S., Blome S., Viltrop A., Penrith M.-L., Chenais E. (2019). Lack of Evidence for Long Term Carriers of African Swine Fever Virus—A Systematic Review. Virus Res..

[13] Lai D.C., Oh T., Nguyen H.T., Do D.T. (2022). The Study of Antigen Carrying and Lesions Observed in Pigs That Survived Post African Swine Fever Virus Infection. Trop. Anim. Health Prod..

[14] Urbano A.C., Ferreira F. (2022). African Swine Fever Control and Prevention: An Update on Vaccine Development. Emerg. Microbes Infect..

[15] European Commission Guidelines on Surveillance and Control of African Swine Fever in Feral Pigs and Preventive Measures for Pig Holdings 2014. https://food.ec.europa.eu/system/files/2016-10/ad_control-measures_asf_wrk-doc-sanco-2013-7138.pdf.

[16] Muñoz-Pérez C., Jurado C., Sánchez-Vizcaíno J.M. (2021). African Swine Fever Vaccine: Turning a Dream into Reality. Transbound. Emerg. Dis..

[17] Coelho Cruz B., Toussaint B., Munoz Pinero A., Mėhn D., Ruiz Moreno A., Van Den Eede G. (2023). JRC Technical Report: African Swine Fever (ASF) Vaccine Development: Progress and Challenges 2023.

[18] Bosch-Camós L., López E., Rodriguez F. (2020). African Swine Fever Vaccines: A Promising Work Still in Progress. Porc. Health Manag..

[19] AVAC AVAC ASF LIVE. http://www.avac.com.vn/en/products-for-pigs/avac-asf-live/.

[20] Borca M.V., Ramirez-Medina E., Silva E., Vuono E., Rai A., Pruitt S., Espinoza N., Velazquez-Salinas L., Gay C.G., Gladue D.P. (2021). ASFV-G-∆I177L as an Effective Oral Nasal Vaccine against the Eurasia Strain of Africa Swine Fever. Viruses.

[21] Ramirez-Medina E., Vuono E., Rai A., Pruitt S., Espinoza N., Velazquez-Salinas L., Pina-Pedrero S., Zhu J., Rodriguez F., Borca M.V. (2022). Deletion of E184L, a Putative DIVA Target from the Pandemic Strain of African Swine Fever Virus, Produces a Reduction in Virulence and Protection against Virulent Challenge. J. Virol..

[22] Ramirez-Medina E., Vuono E., Silva E., Rai A., Valladares A., Pruitt S., Espinoza N., Velazquez-Salinas L., Borca M.V., Gladue D.P. (2022). Evaluation of the Deletion of MGF110-5L-6L on Swine Virulence from the Pandemic Strain of African Swine Fever Virus and Use as a DIVA Marker in Vaccine Candidate ASFV-G-ΔI177L. J. Virol..

[23] Gallardo C., Soler A., Rodze I., Nieto R., Cano-Gómez C., Fernandez-Pinero J., Arias M. (2019). Attenuated and Non-haemadsorbing (Non-HAD ) Genotype II African Swine Fever Virus (ASFV) Isolated in Europe, Latvia 2017. Transbound. Emerg. Dis..

[24] Barasona J.A., Gallardo C., Cadenas-Fernández E., Jurado C., Rivera B., Rodríguez-Bertos A., Arias M., Sánchez-Vizcaíno J.M. (2019). First Oral Vaccination of Eurasian Wild Boar Against African Swine Fever Virus Genotype II. Front. Vet. Sci..

[25] Tamás V., Righi C., Mészáros I., D’Errico F., Olasz F., Casciari C., Zádori Z., Magyar T., Petrini S., Feliziani F. (2023). Involvement of the MGF 110-11L Gene in the African Swine Fever Replication and Virulence. Vaccines.

[26] Petrini S., Righi C., Mészáros I., D’Errico F., Tamás V., Pela M., Olasz F., Gallardo C., Fernandez-Pinero J., Göltl E. (2023). The Production of Recombinant African Swine Fever Virus Lv17/WB/Rie1 Strains and Their In Vitro and In Vivo Characterizations. Vaccines.

[27] Van den Born E., Arias Neira M.L., Gallardo Frontaura C., Fernández Piñero J., Zádori Z., Mészáros I., Olasz F., Sánchez-Vizcaíno J.M., Barroso Arévalo S., Barasona García-Arévalo J.Á. (2022). Attenuated African Swine Fever Virus and Use Thereof in Vaccine Compositions. European Patent.

[28] Gallardo C., Mészáros I., Soler A., Fernandez-Pinero J., van den Born E., Simón A., Casado N., Nieto R., Perez C., Aldea I. (2024). Double Deletion of EP402R and EP153R in the Attenuated Lv17/WB/Rie1 African Swine Fever Virus (ASFV) Enhances Safety, Provides DIVA Compatibility, and Confers Complete Protection Against Genotype II Virulent Strain. Vaccines.

[29] Malogolovkin A., Burmakina G., Tulman E.R., Delhon G., Diel D.G., Salnikov N., Kutish G.F., Kolbasov D., Rock D.L. (2015). African Swine Fever Virus CD2v and C-Type Lectin Gene Loci Mediate Serological Specificity. J. Gen. Virol..

[30] Burmakina G., Malogolovkin A., Tulman E.R., Xu W., Delhon G., Kolbasov D., Rock D.L. (2019). Identification of T-Cell Epitopes in African Swine Fever Virus CD2v and C-Type Lectin Proteins. J. Gen. Virol..

[31] Galindo I., Almazán F., Bustos M.J., Viñuela E., Carrascosa A.L. (2000). African Swine Fever Virus EP153R Open Reading Frame Encodes a Glycoprotein Involved in the Hemadsorption of Infected Cells. Virology.

[32] Alejo A., Matamoros T., Guerra M., Andrés G. (2018). A Proteomic Atlas of the African Swine Fever Virus Particle. J. Virol..

[33] Pei Y., Hodgins D.C., Wu J., Welch S.-K.W., Calvert J.G., Li G., Du Y., Song C., Yoo D. (2009). Porcine Reproductive and Respiratory Syndrome Virus as a Vector: Immunogenicity of Green Fluorescent Protein and Porcine Circovirus Type 2 Capsid Expressed from Dedicated Subgenomic RNAs. Virology.

[34] Kumari S., Chaudhari J., Huang Q., Gauger P., De Almeida M.N., Liang Y., Ly H., Vu H.L.X. (2022). Immunogenicity and Protective Efficacy of a Recombinant Pichinde Viral-Vectored Vaccine Expressing Influenza Virus Hemagglutinin Antigen in Pigs. Vaccines.

[35] Gladue D.P., O’Donnell V., Ramirez-Medina E., Rai A., Pruitt S., Vuono E.A., Silva E., Velazquez-Salinas L., Borca M.V. (2020). Deletion of CD2-Like (CD2v) and C-Type Lectin-Like (EP153R) Genes from African Swine Fever Virus Georgia-∆9GL Abrogates Its Effectiveness as an Experimental Vaccine. Viruses.

[36] Lopez E., Bosch-Camós L., Ramirez-Medina E., Vuono E., Navas M.J., Muñoz M., Accensi F., Zhang J., Alonso U., Argilaguet J. (2021). Deletion Mutants of the Attenuated Recombinant ASF Virus, BA71ΔCD2, Show Decreased Vaccine Efficacy. Viruses.

[37] Petrovan V., Rathakrishnan A., Islam M., Goatley L.C., Moffat K., Sanchez-Cordon P.J., Reis A.L., Dixon L.K. (2022). Role of African Swine Fever Virus Proteins EP153R and EP402R in Reducing Viral Persistence in Blood and Virulence in Pigs Infected with BeninΔDP148R. J. Virol..

[38] Borca M.V., O’Donnell V., Holinka L.G., Sanford B., Azzinaro P.A., Risatti G.R., Gladue D.P. (2017). Development of a Fluorescent ASFV Strain That Retains the Ability to Cause Disease in Swine. Sci. Rep..

[39] Huang L., Liu H., Ye G., Liu X., Chen W., Wang Z., Zhao D., Zhang Z., Feng C., Hu L. (2023). Deletion of African Swine Fever Virus (ASFV) H240R Gene Attenuates the Virulence of ASFV by Enhancing NLRP3-Mediated Inflammatory Responses. J. Virol..

[40] Carrascosa A.L., Bustos M.J., De Leon P. (2011). Methods for Growing and Titrating African Swine Fever Virus: Field and Laboratory Samples. CP Cell Biol..

[41] Gallardo C., Sánchez E.G., Pérez-Núñez D., Nogal M., De León P., Carrascosa Á.L., Nieto R., Soler A., Arias M.L., Revilla Y. (2018). African Swine Fever Virus (ASFV) Protection Mediated by NH/P68 and NH/P68 Recombinant Live-Attenuated Viruses. Vaccine.

[42] Fresco-Taboada A., García-Durán M., Aira C., López L., Sastre P., Van Der Hoek L., Van Gils M.J., Brouwer P.J.M., Sanders R.W., Holzer B. (2022). Diagnostic Performance of Two Serological Assays for the Detection of SARS-CoV-2 Specific Antibodies: Surveillance after Vaccination. Diagn. Microbiol. Infect. Dis..

[43] Villalba M., Lopez L., Redrado M., Ruiz T., de Aberasturi A.L., de la Roja N., Garcia D., Exposito F., de Andrea C., Alvarez-Fernandez E. (2017). Development of Biological Tools to Assess the Role of TMPRSS4 and Identification of Novel Tumor Types with High Expression of This Prometastatic Protein. Histol. Histopathol..

[44] WOAH Terrestrial Animal Health Code 2024. https://www.woah.org/en/what-we-do/standards/codes-and-manuals/terrestrial-code-online-access.

[45] Zhao K., Shi K., Zhou Q., Xiong C., Mo S., Zhou H., Long F., Wei H., Hu L., Mo M. (2022). The Development of a Multiplex Real-Time Quantitative PCR Assay for the Differential Detection of the Wild-Type Strain and the MGF505-2R, EP402R and I177L Gene-Deleted Strain of the African Swine Fever Virus. Animals.

[46] Velazquez-Salinas L., Ramirez-Medina E., Rai A., Pruitt S., Vuono E.A., Espinoza N., Gladue D.P., Borca M.V. (2021). Development Real-Time PCR Assays to Genetically Differentiate Vaccinated Pigs From Infected Pigs With the Eurasian Strain of African Swine Fever Virus. Front. Vet. Sci..

[47] Gladue D.P., Borca M.V. (2022). Recombinant ASF Live Attenuated Virus Strains as Experimental Vaccine Candidates. Viruses.

[48] Borca M.V., Ramirez-Medina E., Espinoza N., Rai A., Spinard E., Velazquez-Salinas L., Valladares A., Silva E., Burton L., Meyers A. (2024). Deletion of the EP402R Gene from the Genome of African Swine Fever Vaccine Strain ASFV-G-∆I177L Provides the Potential Capability of Differentiating between Infected and Vaccinated Animals. Viruses.

[49] Wang L., Fu D., Tesfagaber W., Li F., Chen W., Zhu Y., Sun E., Wang W., He X., Guo Y. (2022). Development of an ELISA Method to Differentiate Animals Infected with Wild-Type African Swine Fever Viruses and Attenuated HLJ/18-7GD Vaccine Candidate. Viruses.

[50] Muñoz A.L., Tabarés E. (2022). Characteristics of the Major Structural Proteins of African Swine Fever Virus: Role as Antigens in the Induction of Neutralizing Antibodies. A Review. Virology.

[51] Kollnberger S.D., Gutierrez-Castañeda B., Foster-Cuevas M., Corteyn A., Parkhouse R.M.E. (2002). Identification of the Principal Serological Immunodeterminants of African Swine Fever Virus by Screening a Virus cDNA Library with Antibody. J. Gen. Virol..

[52] Lopera-Madrid J., Osorio J.E., He Y., Xiang Z., Adams L.G., Laughlin R.C., Mwangi W., Subramanya S., Neilan J., Brake D. (2017). Safety and Immunogenicity of Mammalian Cell Derived and Modified Vaccinia Ankara Vectored African Swine Fever Subunit Antigens in Swine. Vet. Immunol. Immunopathol..

[53] Jancovich J.K., Chapman D., Hansen D.T., Robida M.D., Loskutov A., Craciunescu F., Borovkov A., Kibler K., Goatley L., King K. (2018). Immunization of Pigs by DNA Prime and Recombinant Vaccinia Virus Boost To Identify and Rank African Swine Fever Virus Immunogenic and Protective Proteins. J. Virol..

[54] Sauter-Louis C., Conraths F.J., Probst C., Blohm U., Schulz K., Sehl J., Fischer M., Forth J.H., Zani L., Depner K. (2021). African Swine Fever in Wild Boar in Europe—A Review. Viruses.

[55] Zhang Y., Wang Q., Zhu Z., Wang S., Tu S., Zhang Y., Zou Y., Liu Y., Liu C., Ren W. (2023). Tracing the Origin of Genotype II African Swine Fever Virus in China by Genomic Epidemiology Analysis. Transbound. Emerg. Dis..

[56] Gallardo C., Fernández-Pinero J., Arias M. (2019). African Swine Fever (ASF) Diagnosis, an Essential Tool in the Epidemiological Investigation. Virus Res..

[57] Rueda Pérez P., Sastre Antoranz P., Venteo Moreno Á., González García G., Arias Neira M.L., Gallardo C., Fernández Piñero J., Zádori Z., Mészáros I., Olasz F. Method for Differentiating ASFV Infected from ASFV Vaccinated Animals. European Patent Office Application No. PCT/EP2023/082521, 22 November 2022. Not yet published.

[58] Gallardo M.C., de la Torre Reoyo A., Fernández-Pinero J., Iglesias I., Muñoz M.J., Arias M.L. (2015). African Swine Fever: A Global View of the Current Challenge. Porc. Health Manag..

[59] Martínez Avilés M., Bosch J., Ivorra B., Ramos Á.M., Ito S., Barasona J.Á., Sánchez-Vizcaíno J.M. (2023). Epidemiological Impacts of Attenuated African Swine Fever Virus Circulating in Wild Boar Populations. Res. Vet. Sci..

